# Isokinetic assessment of muscle function according to physical activity level and cardiovascular risk in asymptomatic adults aged 20 to 80 years

**DOI:** 10.1590/1414-431X2025e14214

**Published:** 2025-03-03

**Authors:** V.Z. Dourado, M.B. Nascimento, R.C. Navarro, R.P. da Silva, B.B. Gonze, K.M. Guedes, V.T. Lauria, W.O. Vieira, T.L.V.D.P. Ostolin

**Affiliations:** 1 Departamento de Ciência do Movimento Humano, Universidade Federal de São Paulo, Santos, SP, Brasil; 2 Lown Scholars in Cardiovascular Health Program, Harvard T.H. Chan School of Public Health, Boston, MA, USA

**Keywords:** Muscle strength, Reference values, Dynamometry, Accelerometry, Lower limbs, Upper limbs

## Abstract

As limb muscle function is age- and sex-related, both elbow and knee isokinetic muscle functions and their main predictors, such as physical activity level and cardiovascular risk factors, should be determined. We aimed to describe the percentiles of normality of the isokinetic muscle function of the knee and elbow joints. Secondarily, we developed equations to predict muscle function in apparently healthy adults aged 20-80 years, including cardiovascular risk factors. We conducted a cross-sectional study with 1,334 adults. We collected sociodemographic data, self-reported cardiovascular risk, anthropometry, body composition (bioelectrical impedance), moderate-to-vigorous physical activity (MVPA) (triaxial accelerometry), and isokinetic muscle function. Multiple regression analysis was used to develop equations to predict isokinetic muscle function. Percentiles of normality for muscle function were described by sex and age (20-39, 40-59, and >60 years). The models accounted for 49.6-70.9% of the total variability of muscle function, but MVPA and cardiovascular risk slightly influenced the coefficient of determination (additional ΔR^2^=0.003-0.006). Demographic and anthropometric variables were more relevant predictors of isokinetic muscle function (R^2^=0.50-0.70) than MVPA and cardiovascular risk. Even though they correlated with muscle function, cardiovascular risk and MVPA failed to explain the variability of muscle function largely determined by anthropometric and sociodemographic data. The percentile values and equations developed will help in interpreting the isokinetic muscle function and improve its clinical use.

## Introduction

In recent decades, isokinetic dynamometry has gained popularity as the gold standard for evaluating peripheral muscle function in athletes and patients (1-4). This method offers advantages such as controlling movement velocity over a wide range of motion and providing multiple measures like total work and peak torque, leading to more accurate assessments than other techniques (3). However, the need for representative reference values based on cardiovascular risk and demographic factors poses a challenge to the effective interpretation of isokinetic dynamometer data.

Prior studies have shown that the peak of muscle strength is reached at around 30 years of age, remains stable until around 50 and gradually declines, with an accelerated decrease after 60 years (5,6). This decline is more pronounced in men than women, outpacing muscle mass loss. Differences in muscle strength between upper and lower limbs also become more apparent with age, underscoring the importance of standardized reference values across diverse populations (7). Impaired muscle function is closely linked to daily activities and overall functionality, with objectively measured physical activity strongly associated with muscle size and strength (8).

While self-report is commonly used to assess physical activity, it often underestimates actual activity levels (9), highlighting the importance of objective measurements like accelerometers (10). As the prevalence of chronic conditions like diabetes and hypertension is high, understanding the relationship between these conditions and muscle function is crucial. Studies have shown microcirculatory changes and torque-related alterations in individuals with type 2 diabetes, suggesting a link between muscle function and cardiovascular risk factors (Vigitel Brasil 2013, Ministério de Saúde).

The complexity of this relationship underscores the need for multimodal interventions targeting various aspects of muscle function, aging, and chronic conditions (11). Recent reviews have highlighted the association between muscle strength, cardiovascular risk, and mortality, emphasizing the importance of considering these factors in preventive strategies (12-15).

Given the scarcity of reference values for isokinetic dynamometry, particularly for upper limb joints, this study aimed to provide percentiles of reference values for knee and elbow joints. Additionally, it sought to explore the influence of moderate-to-vigorous physical activity and cardiovascular risk factors on muscle function among asymptomatic adults aged 20-80 years. By providing more comprehensive reference values, the present study may improve the interpretation of isokinetic dynamometry data and enhance preventive strategies for maintaining muscle function and overall health.

## Material and Methods

### Study design and participants

The present study evaluated 1,334 adults (534 males and 800 females) aged 20 to 80 years. The participants were selected from the Epidemiology and Human Movement Study (EPIMOV Study) (16). Briefly, the EPIMOV Study was a prospective cohort study that focused on cardiorespiratory, metabolic, and locomotor health outcomes over a short period of time. A convenient sample of participants was recruited through social networks, posters at regional universities, and local print media.

In the EPIMOV Study, the exclusion criteria were use of gaiters, recent respiratory infections, unstable angina at baseline, stable angina, arrhythmias or electrocardiographic abnormalities during exercise testing, and participant refusal. We also excluded participants who self-reported cognitive and mental health disorders. In the present study, we included only participants free of symptomatic chronic cardiorespiratory and locomotor diseases and no lung function impairment assessed by spirometry.

Participants were informed of the possible risks and discomforts related to our study protocol and provided written consent. The Human Research Ethics Committee of the Federal University of São Paulo (number 186.796) approved the study.

### General health screening

Participants were asked about risk factors for cardiovascular disease (e.g., age, family history of cardiovascular disease, smoking, arterial hypertension, dyslipidemia or hypercholesterolemia, diabetes or hyperglycemia, and physical inactivity).

### Anthropometry and bioelectrical impedance

Body mass (kg) and height (m) were obtained using a scale with a stadiometer as previously described (17), i.e., standing still with bare feet, taking a deep breath, and looking straight ahead. Then, the body mass index was calculated (17).

We obtained body composition using a bioelectrical impedance (310e Biodynamics, USA) at ambient temperature (18). Participants were required to avoid ingesting any liquid or food in the previous four hours and not exerting themselves for at least 12 h before the test. In the supine position, arms and legs were positioned at abduction of 30 and 45 degrees, respectively, for collecting impedance and reactance. We calculated lean and fat body masses for healthy individuals using the Kyle et al. (18) equation. Lastly, we registered lean and fat body masses as absolute and relative (kg and %) values for further analysis.

### Isokinetic dynamometry

Muscle function was assessed through an isokinetic dynamometer (Biodex, System 4, Lumex Inc., USA). The dynamometer was calibrated before the assessments. Before the evaluation, we aligned the mechanical axis of rotation of the device with the rotational axis of the assessed joint (elbow and knee) to apply three protocol tests for the measure of isometric force, peak torque, and total work. The femoral condyle or humerus lateral epicondyle of the tested limb was used as the anatomical reference and aligned with the dynamometer rotation axis following the manufacturer's instructions. For knee and elbow joints, participants were evaluated in a standard seated position, with the limb assessed fixed by strips. The isometric peak torque (Nm) was achieved by performing a trial of five repetitions of isometric force at 60° for 5 s with one-minute intervals. Although peak torque (Nm) and total work (kJ) can be assessed at any angular velocity, we used peak torque as a measure of muscle strength by a trial of five movements at 60°/s with a range of motion from 90° to 0°, and after a five-minute rest, participants performed 30 repetitions at 300°/s to record the total work (kJ) as an index of muscle endurance. These three protocols were applied to the knee extensors and flexors (i.e., quadriceps femoris and hamstrings) and elbow flexors and extensors (i.e., biceps brachii, brachialis, brachioradialis, and triceps brachii) of the dominant side under verbal encouragement. All tests were performed under concentric/concentric protocols.

### Accelerometer-based physical activity

The physical activity level was assessed using a previously validated triaxial accelerometer (GT3X ActiGraph, MTI, USA) (19). We instructed participants to wear the device fixed by a belt around the waist in line with the dominant hip during the week, except when sleeping at night or performing water-related activities. We analyzed the data from participants who used the accelerometer for at least four valid days (i.e., at least 10 h of monitoring per day).

The thresholds for habitual physical activity levels were <3.00 metabolic equivalent (METs) or between 100-1951 counts per minute (CPM) for light-intensity physical activity, 3.00-5.99 METs or between 1952-5724 CPM for moderate physical activity, 6.00-8.99 METs or between 5725-9298 CPM for vigorous physical activity, and ≥9.00 METs or >9499 CPM for very vigorous physical activity (20). Participants with <150 min of moderate-to-vigorous physical activity (MVPA) were considered physically inactive (21).

If needed, participants were instructed to record other activity characteristics, such as posture, walking speed, and weightlifting. The 24-h diary was then reviewed.

### Statistical analysis

Statistical analysis was performed using SPSS software, version 23 (IBM, USA). The data were analyzed descriptively and reported as means±SD for continuous variables and frequency and percentage for categorical variables. We performed independent *t*-tests (for continuous values) and chi-squared tests (for categorical variables) to compare males and females.

First, bivariate correlations between the isokinetic assessment of muscle function and the studied variables were assessed using Pearson or Spearman correlation coefficients for normal or non-normal distributions, respectively. This strategy aimed to identify the variables most significantly related to outcomes in a preliminary univariate analysis. We considered variables with P-values less than 0.1 in the univariate analysis for inclusion in the multivariate models. If two or more explanatory variables were strongly correlated, i.e., with collinearity, only one was included in the model. We used variation inflation factor (VIF) values ≥4 to detect collinearity. Then, several stepwise multivariate linear regression models were developed to predict muscle function, including age, sex, weight, height, MVPA, and cardiovascular risk factors. We aimed to investigate the influence of arterial hypertension, diabetes, obesity, smoking, and dyslipidemia on measures of muscle function. Because of the low impact of cardiovascular risk factors detected in the initial approach, regression models were refit including only age, sex, weight, and height. Lastly, the percentiles of reference values for both isokinetic assessments of knee and elbow muscle function were described according to their percentiles as follows: very poor, ≤5th; poor, 6th to 25th; regular, 26th to 50th; good, 51st to 75th; excellent, 76th to 95th; superior, >95th.

We calculated the sample size based on the number of variables of interest for inclusion in the multivariate models for predicting isokinetic assessment of muscle function (e.g., risk factors for cardiovascular diseases, MVPA, and anthropometric and demographic variables), which indicated 150 participants as a minimum. The alpha error probability was set at 5% for all tests.

## Results

From the EPIMOV Study, 1,334 participants were considered eligible and included in the present study. The sample was composed mainly of middle-aged, overweight females. As expected, we found significant differences between males and females for all variables, except for age and smoking status (Table 1).

**Table 1:** General characteristics of the studied sample (n=1334).

	Males (n=534)	Females (n=800)	Total sample (n=1334)
Age (years)	42±14	44±16	42±16
Anthropometry and body composition, mean±SD			
Body mass (kg)*	81.4±15.4	73.1±17.4	76.2±17.2
Height (m)*	1.73±0.07	1.58±0.07	1.64±0.10
Body Mass Index (kg/m^2^)*	27.0±4.6	28.9±6.6	28.2±6.0
Lean body mass (kg)*	61.8±9.4	47.0±8.5	52.6±11.4
Lean body mass (%)*	76.2±7.2	65.0±9.0	69.3±10.0
Fat body mass (kg)*	19.6±8.7	26.2±11.0	23.7±10.7
Fat body mass (%)*	23.5±6.3	34.4±7.1	30.3±8.6
Cardiovascular risk, n (%)			
Arterial hypertension*^€^	49 (9.1)	178 (22.3)	227 (17.0)
Diabetes mellitus*^€^	28 (5.3)	91 (11.4)	119 (8.99)
Obesity*^€^	84 (15.7)	243 (30.4)	332 (24.5)
Dyslipidemia*^€^	108 (19.2)	325 (40.6)	433 (32.5)
Current smoking*^€^	55 (10.4)	107 (13.4)	162 (12.1)
Physical inactivity*^#^	82 (15.3)	216 (27.0)	319 (22.3)
Accelerometer-based physical activity, mean±SD			
Sedentary time (min/week)	3718±913	3755±969	3741±948
Sedentary time (%)	72.5±6.0	72.1±6.0	72.2±6.0
Light-intensity physical activity (min/week)	1112±334	1199±407	1166±383
Light-intensity physical activity (%)*	21.7±5.2	22.9±5.1	22.4±5.2
Moderate physical activity (min/week)*	268±128	243±123	253±126
Moderate physical activity (%)*	5.1 ±2.2	4.5±2.2	4.8±2.2
Vigorous physical activity (min/week)*	21.0±37.7	12.3±21.7	15.6±29.1
Vigorous physical activity (%)*	0.4±0.7	0.3±0.5	0.3±0.6
Very vigorous physical activity (min/week)*	3.7±12.6	1.8±6.5	2.6±9.3
Very vigorous physical activity (%)*	0.1±0.3	0.1±0.2	0.1±0.2
Moderate-to-vigorous physical activity (min/week)*	293±143	257±130	271±136
Moderate-to-vigorous physical activity (min/day)*	49.6±21.2	42.7±19.5	45.3±20.5
Moderate-to-vigorous physical activity (%)*	5.7±2.4	4.9±2.3	5.2±2.4
Isokinetic assessment of muscle function, mean±SD			
Knee extension			
Peak torque at 60°/s (Nm)*	184.7±70.5	110.9±36.0	138.9±63.0
Total work at 300°/s (kJ)*	2192±675	1308±366	1643±684
Isometric peak torque (Nm)*	210.2±61.5	135.6±63.9	163.9±72.6
Knee flexion			
Peak torque at 60°/s (Nm)*	87.8±30.7	49.1±17.8	63.8±30.1
Total work at 300°/s (kJ)*	1287±561	694±370	919±535
Elbow flexion			
Peak torque at 60°/s (Nm)*	42.7±16.2	27.5±13.8	33.2±16.5
Total work at 300°/s (kJ)*	1022±411	755±907	856±769
Isometric peak torque (Nm)*	54.0±33.9	36.1±27.7	42.9±31.4
Elbow extension			
Peak torque at 60°/s (Nm)*	49.0±38.0	34.5±18.0	40.0±28.2
Total work at 300°/s (kJ)*	1062±705	592±449	770±604

Data are reported as means±SD or number (%). *P≤0.05: males *vs* females. Independent *t*-tests (for continuous values) and chi-squared tests (for categorical variables). ^€^Assessed by self-report; ^#^assessed using a triaxial accelerometer.

Regarding physical activity level, male participants also presented higher levels of physical activity in minutes/week and in percentage for all registered intensities (e.g., light, moderate, moderate-to-vigorous, vigorous, and very vigorous intensity). In contrast, we found no differences in sedentary behavior between males and females (Table 1).

Descriptive data of the isokinetic assessment of muscle function of the knee and elbow are also described in Table 1. The data are reported as mean and standard deviation for males, females, and the total sample. As expected, we found higher values for male than for female participants.

We found significant weak correlations between physical activity and isokinetic assessment of muscle function, mainly in the knee. Sedentary time did not correlate with muscle function, except for total work on elbow flexion. Regarding elbow muscle function, the correlations were less consistent than for the knee joint. However, MVPA correlated with all isokinetic assessments of muscle function measures of both elbow and knee joints. Lastly, total work and isometric peak torque of elbow flexion presented a stronger correlation with physical activity than other elbow measurements.

After stepwise multiple linear regression analysis, MVPA was only included for the isometric peak torque of knee extension and flexion. Risk factors for cardiovascular disease were significant predictors of knee extension total work and elbow muscle function (e.g., isometric peak torque and peak torque of extension and flexion). Although the multivariate models explained 49.6 to 70.9% of muscle function variability, MVPA and cardiovascular disease risk factors had little contribution to the coefficient of determination (ΔR^2^=0.003 to 0.006). Therefore, anthropometric and demographic variables explained the variation of isokinetic muscle function of the knee (R^2^=52.5 to 70.9%) and elbow (R^2^=49 to 70.7%), as seen in Tables 2 and 3, respectively.

**Table 2:** Stepwise multiple regression analysis results with moderate-to-vigorous physical activity (MVPA) and risk factors for cardiovascular disease as independent predictors and isokinetic muscle function as the outcome (n=1334).

Isokinetic muscle function	Moderate-to-vigorous physical activity and/or risk factor for cardiovascular disease	β (SE)	R^2^	ΔR^2^	Other significant predictors
Knee extension					
Peak torque at 60°/s (Nm)	-	-	0.697	-	Height; sex; age; body mass
Total work at 300°/s (kJ)	-	-	0.654	-	Height; sex; age; body mass
Isometric peak torque (Nm)	MVPA (min/day)^#^	0.176 (0.058)	0.683	0.004	Height; sex; age; body mass
Knee flexion					
Peak torque at 60°/s (Nm)	MVPA (min/day)^#^	0.076 (0.032)	0.624	0.003	Height; age; sex; body mass
Total work at 300°/s (kJ)	Diabetes	-103.755 (50.992)	0.523	0.003	Sex; age; height
Elbow flexion					
Peak torque at 60°/s (Nm)	Dyslipidemia	1.874 (0.893)	0.709	0.003	Sex; body mass; age; height
Total work at 300°/s (kJ)	-	-	0.565	-	Sex; body mass; age
Isometric peak torque (Nm)	Physical inactivity^#^	-2.413 (1.134)	0.651	0.003	Sex; body mass; age; height
Elbow extension					
Peak torque at 60°/s (Nm)	Obesity	-4.024 (1.469)	0.657	0.006	Sex; body mass; age
Total work at 300°/s (kJ)	-	-	0.496	-	Sex; body mass; age

The models were adjusted for age (years), body mass (kg), height (m), sex (men=1; women=0), moderate-to-vigorous physical activity (min/day), arterial hypertension (yes=1; no=0), diabetes (yes=1; no=0), dyslipidemia (yes=1; no=0), obesity (body mass index ≥30 kg/m^2^=1; body mass index <30 kg/m^2^=0), smoking (yes=1; no=0), and physical inactivity (yes=1; no=0). ^#^Assessed using a triaxial accelerometer.

**Table 3:** Multivariate models to predict isokinetic muscle function in asymptomatic adults based on anthropometric and demographic attributes (n=1334).

Isokinetic muscle function	Constant	Age (years)	Body mass (kg)	Height (m)	Sex^#^	R^2^	Adjusted R^2^	SEE	Lower limit of 95%CI
Knee extension									
Peak torque at 60°/s (Nm)	-127.3	-1.3	0.5	162.1	44.1	0.704	0.702	31.3	51.4
Total work at 300°/s (kJ)	-180.4	-18.0	5.5	1174.6	640.1	0.652	0.650	417.6	686.9
Isometric peak torque (Nm)	-206.7	-1.0	0.5	222.0	43.4	0.678	0.676	34.6	56.9
Knee flexion									
Peak torque at 60°/s (Nm)	-65.5	-0.6	0.1	81.1	26.2	0.621	0.620	19.5	32.0
Total work at 300°/s (kJ)	226.8	-13.5	0.4	626.2	485.3	0.525	0.523	373.4	614.2
Elbow flexion									
Peak torque at 60°/s (Nm)	-10.6	-0.1	0.2	17.1	18.5	0.707	0.705	7.9	12.9
Total work at 300°/s (kJ)	211.5	-4.2	5.2	99.1	359.3	0.670	0.665	207.9	341.9
Isometric peak torque (Nm)	-24.5	-0.2	0.2	29.7	21.3	0.646	0.643	11.0	18.0
Elbow extension									
Peak torque at 60°/s (Nm)	-11.5	-0.2	0.2	21.3	20.9	0.648	0.646	10.3	16.9
Total work at 300°/s (kJ)	499.6	-5.7	5.2	-58.8	553.4	0.569	0.566	328.3	540.0

^#^Factor: sex: males=1; females=0. SEE: standard error of estimate; Lower limit=1.645 × SEE.

Tables 4-7 present the percentiles of the reference values of isokinetic muscle function of the knee and elbow joints according to sex and age ranges (i.e., 20-39, 40-59, and ≥60 years).

**Table 4:** Percentiles of reference values for isokinetic assessment of muscle function of the elbow in females according to age.

Age (years)	Very poor	Poor	Regular	Good	Excellent	Superior
Elbow flexion						
Peak torque at 60°/s (Nm)						
20-39	<14.4	14.4-19.5	19.5-23.1	23.1-27.0	27.0-39.3	>39.3
40-59	<16.7	16.7-20.0	20.0-23.7	23.7-29.4	29.4-37.2	>37.2
≥60	<14.1	14.1-18.2	18.2-22.8	22.8-27.7	27.7-36.5	>36.5
Total work at 300°/s (kJ)						
20-39	<319.8	319.8-436.8	436.8-520.3	520.3-705.2	705.2-1014.3	>1014.3
40-59	<385.9	385.9-480.2	480.2-577.4	577.4-664.8	664.8-869.9	>869.9
≥60	<263.4	263.4-440.2	440.2-532.4	532.4-630.4	630.4-801.2	>801.2
Isometric peak torque (Nm)						
20-39	<17.1	17.1-24.7	24.7-29.1	29.1-35.2	35.2-47.4	>47.4
40-59	<19.0	19.0-24.8	24.8-30.0	30.0-35.2	35.2-47.1	>47.1
≥60	<16.1	16.1-23.2	23.2-28.5	28.5-34.2	34.2-46.3	>46.3
Elbow extension						
Peak torque at 60°/s (Nm)						
20-39	<18.2	18.2-23.0	23.0-28.1	28.1-33.6	33.6-44.8	>44.8
40-59	<17.8	17.8-23.9	23.9-29.3	29.3-35.4	35.4-43.0	>43.0
≥60	<13.5	13.5-21.6	21.6-26.7	26.7-31.6	31.6-41.2	>41.2
Total work at 300°/s (kJ)						
20-39	<187.4	187.4-352.8	352.8-498.3	498.3-635.0	635.0-1019.8	>1019.8
40-59	<176.3	176.3-378.3	378.3-525.9	525.9-704.0	704.0-945.6	>945.6
≥60	<108.5	108.5-310.3	310.3-484.8	484.8-653.1	635.1-956.9	>956.9

Classification according to the percentiles found: very poor, ≤5th; poor, 6th to 25th; regular, 26th to 50th; good, 51st to 75th; excellent, 76th to 95th; superior, >95th.

**Table 5 t05:** Percentiles of reference values for isokinetic assessment of muscle function of the elbow in males according to age.

Age (years)	Very poor	Poor	Regular	Good	Excellent	Superior
Elbow flexion						
Peak torque at 60°/s (Nm)						
20-39	<34.0	34.0-42.9	42.9-48.7	48.7-60.8	60.8-74.8	>74.8
40-59	<31.1	31.1-41.9	41.9-46.4	46.4-55.1	55.1-69.7	>69.7
≥60	<23.9	23.9-35.3	35.3-40.0	40.0-45.1	45.1-54.8	>54.8
Total work at 300°/s (kJ)						
20-39	<679.1	679.1-871.1	871.1-1040.0	1040.0-1241.3	1241.3-1578.8	>1578.8
40-59	<626.3	626.3-846.9	846.9-1000.3	1000.3-1219.0	1219.0-1518.3	>1518.3
≥60	<376.4	376.4-660.1	660.1-819.5	819.5-980.1	980.1-1334.2	>1334.2
Isometric peak torque (Nm)						
20-39	<38.3	38.3-51.5	51.5-63.4	63.4-71.7	71.7-98.1	>98.1
40-59	<22.5	22.5-48.3	48.3-58.8	58.8-66.2	66.2-86.4	>86.4
≥60	<30.9	30.9-42.1	42.1-51.4	51.4-59.7	59.7-71.7	>71.7
Elbow extension						
Peak torque at 60°/s (Nm)						
20-39	<33.6	33.6-48.6	48.6-58.4	58.4-70.4	70.4-89.0	>89.0
40-59	<32.9	32.9-48.0	48.0-55.5	55.5-64.9	64.9-89.7	>89.7
≥60	<26.0	26.0-37.8	37.8-45.6	45.6-53.9	53.9-63.0	>63.0
Total work at 300°/s (kJ)						
20-39	<551.8	551.8-913.3	913.3-1241.0	1241.0-1562.4	1562.4-2026.3	>2026.3
40-59	<346.3	346.3-944.9	944.9-1149.0	1149.0-1441.9	1441.9-1946.6	>1946.6
≥60	<299.5	299.5-619.4	619.4-1018.7	1018.7-1172.8	1172.8-1658.4	>1658.4

Classification according to the percentiles found: very poor, ≤5th; poor, 6th to 25th; regular, 26th to 50th; good, 51st to 75th; excellent, 76th to 95th; superior, >95th.

**Table 6 t06:** Percentiles of reference values for isokinetic assessment of knee muscle function in females according to age.

Age (years)	Very poor	Poor	Regular	Good	Excellent	Superior
Knee extension						
Peak torque at 60°/s (Nm)						
20-39	<87.5	87.5-111.5	111.5-127.7	127.7-150.3	150.3-186.1	>186.1
40-59	<62.7	62.7-93.0	93.0-119.8	119.8-138.5	138.5-169.1	>169.1
≥60	<42.9	42.9-73.4	73.4-90.2	90.2-107.5	107.5-135.7	>135.7
Total work at 300°/s (kJ)						
20-39	<979.9	979.9-1262.6	1262.6-1530.0	1530.0-1795.4	1795.4-2225.9	>2225.9
40-59	<792.0	792.0-1080.4	1080.4-1314.7	1314.7-1586.1	1586.1-1925.8	>1925.8
≥60	<513.0	513.0-808.5	808.5-1068.8	1068.8-1290.6	1290.6-1631.7	>1631.7
Isometric peak torque (Nm)						
20-39	<100.8	100.8-132.4	132.4-153.6	153.6-177.0	177.0-216.1	>216.1
40-59	<88.9	88.9-115.4	115.4-142.0	142.0-168.6	168.6-199.1	>199.1
≥60	<64.6	64.6-95.9	95.9-116.9	116.9-138.7	138.7-167.0	>167.0
Knee flexion						
Peak torque at 60°/s (Nm)						
20-39	<35.4	35.4-49.1	49.1-60.2	60.2-69.4	69.4-89.7	>89.7
40-59	<14.5	14.5-38.2	38.2-51.0	51.0-63.1	63.1-78.3	>78.3
≥60	<17.1	17.1-30.4	30.4-39.8	39.8-49.1	49.1-63.5	>63.5
Total work at 300°/s (kJ)						
20-39	<269.8	269.8-631.7	631.7-890.1	890.1-1050.9	1050.9-1450.8	>1450.8
40-59	<168.3	168.3-436.8	436.8-677.5	677.5-901.5	901.5-1203.1	>1203.1
≥60	<55.9	55.9-298.0	298.0-449.1	449.1-632.0	632.0-892.3	>892.3

Classification according to the percentiles found: very poor, ≤5th; poor, 6th to 25th; regular, 26th to 50th; good, 51st to 75th; excellent, 76th to 95th; superior, >95th.

**Table 7:** Percentiles of reference values for isokinetic assessment of knee muscle function in males according to age.

Age (years)	Very poor	Poor	Regular	Good	Excellent	Superior
Knee extension						
Peak torque at 60°/s (Nm)						
20-39	<137.5	137.5-181.7	181.7-212.9	212.9-241.1	241.1-289.1	>289.1
40-59	<107.2	107.2-161.6	161.6-186.4	186.4-219.9	219.9-271.2	>271.2
≥60	<80.9	80.9-108.8	108.8-131.7	131.7-159.8	159.8-205.5	>205.5
Total work at 300°/s (kJ)						
20-39	<1586.5	1586.5-2284.1	2284.1-2534.8	2534.8-2982.7	2982.7-3520.4	>3520.4
40-59	<1226.4	1226.4-1792.0	1792.0-2201.8	2201.8-2572.6	2572.6-3076.1	>3076.1
≥60	<755.4	755.4-1275.1	1275.1-1525.9	1525.9-1853.5	1853.5-2378.7	>2378.7
Isometric peak torque (Nm)						
20-39	<161.8	161.8-201.5	201.5-242.5	242.5-267.7	267.7-327.0	>327.0
40-59	<141.4	141.4-193.1	193.1-227.4	227.4-248.6	248.6-302.7	>302.7
≥60	<105.9	105.9-150.7	150.7-175.8	175.8-200.0	200.0-266.0	>266.0
Knee flexion						
Peak torque at 60°/s (Nm)						
20-39	<57.7	57.7-86.7	86.7-105.6	105.6-124.5	124.5-144.3	>144.3
40-59	<48.1	48.1-73.1	73.1-91.5	91.5-108.3	108.3-134.2	>134.2
≥60	<21.5	21.5-52.6	52.6-65.4	65.4-81.2	81.2-105.5	>105.5
Total work at 300°/s (kJ)						
20-39	<820.3	820.3-1240.0	1240.0-1518.5	1518.5-1875.0	1875.0-2447.4	>2447.4
40-59	<351.1	351.1-891.0	891.0-1277.3	1277.3-1610.2	1610.2-2081.6	>2081.6
≥60	<147.7	147.7-413.1	413.1-797.2	797.2-1154.5	1154.5-1505.8	>1505.8

Classification according to the percentiles found: very poor, ≤5th; poor, 6th to 25th; regular, 26th to 50th; good, 51st to 75th; excellent, 76th to 95th; superior, >95th.

## Discussion

In the present study, we described the percentiles of reference values of knee and elbow isokinetic muscle function in asymptomatic adults stratified according to sex and age. The literature lacks these values in asymptomatic adults, especially considering the influence of accelerometer-based physical activity levels and cardiovascular risk. In addition to providing reference values, our results showed that anthropometric and sociodemographic variables are the main predictors of isokinetic muscle function, even when adjusted for cardiovascular risk and physical activity level. Our sample was primarily composed of middle-aged females who were overweight, which is similar to what is observed in the Brazilian population (e.g., the prevalence of overweight is 55.4%) (Vigitel database). The cardiovascular risk was higher in females than in males.

Although significant, weak correlations were found between physical activity and isokinetic assessment of muscle function, mainly for knee muscle function. Thus, our findings corroborate a recent systematic review that found only a mild positive correlation between objective physical activity and muscle function (7). Additionally, our results agreed with another recent systematic review and meta-analysis (22), which showed more significant associations between physical activity and muscle function in the lower limb than in the upper limb. MVPA consistently correlated with the isokinetic muscle function of both the elbow and knee, reinforcing previous results (22). It is worth noting that upper limb function is usually associated with measurements of handgrip strength, which cannot be used for further comparisons since we analyzed isokinetic muscle function of the elbow.

Physical inactivity was not associated with muscle function, except for the total work of elbow extension. While physical inactivity has several negative effects, it does not significantly impact muscle strength until there is severe muscle mass loss (23). Previous research by Yatsugi et al. (24) found that MVPA was linked to physical function measures like handgrip strength, walking speed, and chair-standing time, but not with light-intensity activity or sedentary time. The relationship between physical inactivity and muscle function may depend on the total amount of MVPA and sedentary behavior (22,25). Additionally, as people age, physical inactivity increases, potentially affecting daily living activities. Samuel et al. (26) noted that lower limb strength tends to decline more than upper limb strength with physical inactivity.

Similar to our findings, Saito et al. (27) evaluated 4,249 middle-aged Japanese subjects and found negative correlations between muscle function and smoking status. Nevertheless, this association was attenuated or became non-significant after adjusting for body mass, height, and age. In contrast, obesity tends to increase strength and power due to weight-bearing, but its influence is reduced when adjusting for body mass and is linked to specific muscle groups (28). Blakeley et al. (29) observed an association between dyslipidemia and high handgrip strength in children, but it was not independent of the BMI z-score. These findings reinforce the importance of the anthropometric and demographic attributes and help to understand possible differences regarding upper and lower limb strength, despite methodological differences of the studies.

Our results also agree with Lee et al. (30), who evaluated 8,208 participants and found an association between muscle function and type 2 diabetes (i.e., handgrip strength and insulin resistance). In a narrative review, Nomura et al. (31) discussed the effects and associations between diabetes and muscle mass and strength in lower limbs, highlighting the role of diabetic neuropathy that accelerates motor dysfunction. However, Akpinar et al. (32) did not observe an association of being diabetic and time after diagnosis with muscle strength or muscle mass, which can suggest that controlled diabetes does not necessarily accelerate the decrease in muscle mass and strength. Like Akpinar et al. (32), our participants presented a high cardiovascular risk but without signs or symptoms of uncontrolled conditions that could compromise the capability to perform the effort. In addition, patients with diabetes had decreased muscle strength, but the severity of diabetic neuropathy is related only to proximal muscle groups (32,33). We can attribute these results to characteristics of the participants, since they were primarily middle-aged and healthy or with treated and controlled chronic conditions. Therefore, these variables did not improve the coefficient of determination. Hence, our equations based on anthropometric and demographic attributes combined with the percentiles of reference values are sufficient to identify individuals with reduced peripheral muscle function.

Our study presented limitations that must be considered. The convenience sample is one of our main limitations. However, our sample was similar to the Brazilian population (Vigitel 2013), which can minimize this possible bias. Although the sample size was sufficient to carry out the proposed analysis, the higher proportion of middle-aged subjects allowed us to only stratify the sample into three age groups (e.g., 20-39, 40-59, and ≥60 years) instead of using ten-year age groups. Nevertheless, we are confident about the generalizability of our findings, mainly due to our detailed study protocol that included electrical bioimpedance, triaxial accelerometry, cardiopulmonary exercise testing, and cardiovascular risk assessment. Finally, the method used in the present study to assess the level of physical activity, although considered ideal for epidemiological studies of this dimension, suffers from its inability to measure strengthening, for example. This may have compromised the correlations between the level of physical activity and muscle function found in the present study.

Although MVPA was included in the models developed for predicting the isometric peak torque of knee extension and knee flexion, its inclusion did not affect the overall predictive ability (ΔR^2^=0.003 to 0.006), corroborating previous studies (34,35). Based on an isokinetic assessment of muscle function similar to ours, Pereira et al. (35) proposed a prediction equation for peak torque of knee extension using age, body mass, and height (R^2^=0.30). Our results were similar to Neder et al. (34) models for knee muscle function. In contrast, our multivariate models explained 49.6 to 70.9% of knee and elbow muscle function variability. It is essential to consider that our models based on anthropometric and demographic attributes largely determined muscle function in agreement with previous findings of strong correlations between muscle function, age, sex, height, and body mass (36). To our knowledge, the literature still lacks predictive models for elbow muscle function, which is one of the main practical implications of the present study.

Monitoring muscle function is also essential since muscle strength is associated with all-cause mortality (37,38), regardless of lean body mass. Accordingly, our percentiles of reference values will contribute greatly in the assessment of muscle function over time. In addition, we provided the main predictors of muscle function for both upper and lower limbs, which helps design preventive strategies to increase or maintain muscle strength and early identification of compromised muscle function. Despite the practical implications of our findings, we are interested in further complementing our data based on analysis with ten-year age groups instead of the wide range used in the present study.

## Conclusions

Physical activity had low predictive value for muscle function despite its correlation with the isokinetic muscle function of both knee and elbow joints. Similarly, risk factors for cardiovascular disease presented little or no influence on peripheral muscle function. In contrast, anthropometric and demographic attributes were important predictors of muscle function in asymptomatic adults. Therefore, the developed equations based on age, sex, body mass, and height combined with the percentiles of reference values can contribute to the best assessment of isokinetic muscle function. Furthermore, our findings may help expand the use of the isokinetic dynamometer in clinical practice, instead of often limiting it to patients undergoing rehabilitation and athletes.
